# Comparative genomics of human brain and immune gene preservation across species

**DOI:** 10.1371/journal.pone.0348713

**Published:** 2026-05-11

**Authors:** Xiao Liang, Andrew F. Teich, Lenwood S. Heath

**Affiliations:** 1 Department of Pathology and Cell Biology, Columbia University, New York, New York, United States of America; 2 Department of Computer Science, Virginia Polytechnic Institute and State University, Blacksburg, Virginia, United States of America; 3 Department of Neurology, Columbia University, New York, New York, United States of America; 4 Taub Institute for Research on Alzheimer’s Disease and the Aging Brain, Columbia University, New York, New York, United States of America; University of Massachusetts, UNITED STATES OF AMERICA

## Abstract

The study of human gene evolution along primate and non-primate lineages has attracted increasing attention. Previous research demonstrated associations between the origin of genes and their expression in various tissues, including human-specific genes contributing to the brain. However, the relationship between gene tissue expression and their existence in evolutionary history has rarely been systematically examined from a phylogenetic perspective. In this study, we analyzed 1360 human genes highly expressed in the brain and/or the immune system, along with their distribution in 31 non-human primate species and 4 non-primate species. Two control sets were included for comparison: a randomly selected set of 295 human genes, and a set of 369 human genes each representing a distinct HGNC gene family. We discovered that compared to random and immune-related genes (genes highly expressed in immune system), brain genes (genes highly expressed in brain) have earlier origins, predating primates, and have been preserved across various primate species. We also show that these earlier origins are unlikely to be due to genes that are widely highly expressed in many tissues. Moreover, genes highly expressed in both the brain and immune system display a tendency toward early origin, consistent with other brain genes. This observation indicates that genes highly expressed in both systems are more likely to begin with high expression in the brain, subsequently acquiring high expression in immune tissues, rather than vice versa. We investigated the brain and immune-related genes that are estimated to have emerged among primates, as well as genes that originated before primates but are absent in certain primate clades. Genes in the latter group were either highly expressed across more than ten tissues or specifically expressed in no more than two organ systems, suggesting that these genes may be either broadly essential or highly specialized, performing specific functions in a few systems.

## Introduction

The evolutionary history of genes from different species, particularly in relation to human genes, offers a compelling field of study. Numerous studies have investigated the primate and human specific genes. Some of these studies have focused on their possible evolutionary origins within the primates and from ancestral species [1–9], whereas some others have examined their characteristics [8,10–17].

While early studies have focused on gene duplication, a long-studied mechanism in gene evolution, a more recent proposed mechanism is that some genes specific to a species possibly originate from previously noncoding regions. This mechanism has been supported by more and more evidence in a variety of species, including yeast [1], nematode [2], fruit fly [3,4], mouse [5], hominoids [6–8], and human [9], highlighting the emergence of new genes in specific species while being absent in others.

Other research has identified associations between primate and human specific genes with particular tissues and diseases. For instance, the human specific genes *ARHGAP11B* and *NOTCH2NL* are reported to be associated with neocortical expansion in the human brain [10–14]. Primate and human specific genes are also found to have involved in adaptations affecting the immune system, along with brain and metabolism [18].

Previous work has investigated the loss of genes and their functions [19–22]. However, this area has been less thoroughly explored across a wide range of species. Broadening the scope of this research could unveil a deeper understanding of the evolutionary significance of gene presence and absence and the correlation with gene expression in diverse tissues.

The brain and the immune system represent crucial areas of study within the human species, attracting attention in recent research. There has been abundant prior work on the evolution of the primate brain studied from various perspectives [23–26]. One important topic among those is brain size in primates. Previous studies have shown that the brain size volume and cortical folding have increased in recently diverged primates, such as hominoids, compared to older primates [23,24]. Furthermore, *Hominoidea* and *Cercopithecinae* evolved their brain shape more rapidly than *Strepsirrhini*, *Colobinae*, and *Platyrrhini* [24]. In contrast, the human brain demonstrates more evolutionary constraint in gene expression than other organs compared to non-human primates [25,26]. Rogers et al. [23] suggest that the observed correlation between brain size and cortical folding cannot be solely attributed to one set of selective pressures or genetic changes. Multiple attempts have been made to connect brain-expressed genes and brain characteristics among primates, but it remains a challenging topic today [25].

In this study, we aim to explore the existence and distribution patterns of human genes that are highly expressed in the brain and the immune system, both in non-human primates and in non-primate species. This investigation emphasizes the distribution of genes across various species and offers potential insights into evolutionary processes through their origin, preservation, and absence in different species. Our study examined the distribution of 1360 human genes across 32 primate species (including human) and 4 non-primate species. Two control gene sets are included for comparison purposes: a randomly selected set of 295 human genes, and a set of 369 human genes each from a distinct HUGO Gene Nomenclature Committee (HGNC) [27] gene family group.

In this study, we use the term brain genes to refer to genes highly expressed in the brain, without requiring that such expression be exclusive to brain tissue, while the term immune-related genes refer to genes highly expressed in the immune system. Note that the definition can be different from other literature.

The results indicate that brain genes are more preserved than immune-related genes and random genes, which is consistent with previous studies [26,28,29]. On this basis, we observed that genes highly expressed in both the brain and immune system are more likely to acquire high brain expression earlier than high immune expression, as their existence across non-human species tends to align with that of other brain genes rather than immune-related ones. Analyses of genes either broadly or specifically highly expressed in certain tissues indicate that the latter is more likely to contribute to the earlier origin time of brain genes rather than the former.

We have also identified a potential set of brain and immune-related genes that emerged in primates. Our results demonstrate that these genes are either broadly expressed in at least ten tissues (whereas the median number of tissues in which all genes are highly expressed is three), or almost specifically in the brain. For the potential set of brain and immune-related genes that predated primates but were lost in certain primate clades, our findings show that these genes are either broadly expressed in more than 15 tissues or specifically in the cerebellum or cerebral cortex. These patterns hint a possible relationship between the evolutionary origin of genes and their functional expression profiles, indicating that genes with essential roles across multiple tissues or highly specialized functions may be evolutionarily resilient across most primate species.

## Materials

There are six types of data used in this work: (1) CDS (coding sequence) data of 32 primate species and 4 non-primate species [30,31]; (2) tissue specific gene expression data obtained from the Human Protein Atlas 23.0 [32,33]; (3) 1360 human genes highly expressed in the brain or the immune system [32,33]; (4) 295 random human genes selected using the random.sample() function in Python 3.10.8; (5) 369 human genes each from a distinct HGNC [27] gene family group; and (6) protein sequences of the genes obtained from UniProt [34].

### Coding sequences of primate and non-primate species

In this research, we examined the genomes and coding sequences (CDS) of 32 primate species, including *Homo sapiens*, along with four non-primate species, utilizing data from the Ensembl and NCBI databases [30,31]. The 32 primate species are all primates with whole-genome sequences available on Ensembl or NCBI databases at the time we perform the investigation.

Non-primate species investigated in this study include mouse, dog, zebrafish, and anteater. The genome accession numbers used in this analysis are detailed in Data Availability. The first three species are established model organisms studied in prior work [35]. Anteater was additionally included as a lesser-studied, endemic species from the New World. It should be noted that the anteater genome is not as well-annotated as the other three species, and the results reported for anteaters are rather supplementary than essential.

### Tissue specific gene expression

The gene expression data in different human tissues were obtained from the Human Protein Atlas [32,33]. Specifically, normal tissue data were used, which comprises protein expression profiles in human tissues obtained via immunohistochemistry with tissue microarrays, based on the Human Protein Atlas version 23.0 and Ensembl version 109.

### Human genes highly expressed in brain and immune system

From the expression data in normal human tissues, we have selected genes that have both “High” expression level and “Enhanced” reliability score, both obtained from the normal tissue expression data [32,33], in the following primary organ tissues: (1) brain tissues: caudate, cerebellum, cerebral cortex, hippocampus, hypothalamus, dorsal raphe, substantia nigra; and (2) immune system tissues: bone marrow, thymus, and lymph node. A complete list of tissues on Human Protein Atlas version 23.0 [32,33] is provided in the GitHub repository in Data Availability. Note that we have selected these primary organ tissues with the intention to study tissues that are most directly related to neural and immune cell activities. Therefore, the findings and conclusions of this study are based solely on the analysis of these tissues and may not be applicable when more broadly associated tissues are considered..

Here, the expression levels provided in the original data from Human Protein Atlas [32,33] have four categorical values: “High,” “Medium,” “Low,” and “Not detectable.” An “Enhanced” reliability score is assigned to genes for which an antibody or several antibodies, targeting non-overlapping sequences of the same gene, have received enhanced validation through orthogonal or independent antibody validation methods [32,33]. In this study, we only consider genes with a “High” expression level and “Enhanced” reliability score to be highly expressed genes for later investigation. This selection process resulted in a total of 1403 distinct genes, with 1055 genes highly expressed in the brain, 605 genes in the immune system, and 257 overlap genes in both types of tissues.

For all the 1403 human genes examined, there are 40 genes not detected in any of the 36 species using the BLAST method described in Section Methods. The non-detection of these genes in the 36 species examined may not necessarily indicate their actual absence but may be attributed to the sensitivity thresholds or limitation of the methods and genome sequences. These genes were excluded from later analysis.

In addition, there are three pairs of genes in which each pair is mapped to the identical protein: {*ATP2B2*, *ATP2B4*}, {*HBA1*, *HBA2*}, and {*MCM2*, *MCM7*}. In the analysis, we only kept the first gene from each pair, adhering to the presented order.

In the remainder of the paper, we will denote by ℬ the set of 1019 genes highly expressed in human brain tissues, by ℐ the 586 genes highly expressed in the human immune system, and by 𝒞=B∩I the 245 genes highly expressed both in the human brain and immune system, respectively. Genes not detected in any species have already been excluded from these sets. In total, 1360 genes have been processed in further analysis.

To assess whether the genes highly expressed in brain tissues and immune-related tissues were functionally related to their respective tissue categories, we performed over-representation analysis (ORA) for each gene set separately using Gene Ontology (GO) terms and Reactome pathways. These analyses was used to examine whether brain-related and immune-related annotations were enriched in ℬ and ℐ, respectively. The observed enrichment profiles were broadly in agreement with the expected brain and immune-related functions, supporting the biological relevance of these gene sets. The corresponding results are presented in Supplementary Fig S1 in S1 File.

### Random human genes

In this study, we have examined a union of random human gene sets used in our previous papers [36,37], randomly selected using the random.sample() function in Python 3.10.8 from all the human coding sequences (Genome Reference Consortium Human Build 38) obtained from Ensembl [30]. After excluding genes that overlap with those highly expressed in the brain and immune system, the resulting set comprised 304 genes. Out of the 304 genes, 9 genes that are not identified in any species, including human, were excluded from further analysis.

Ideally, the control set can be all the other genes in human genome. However, With resource and processing limitations on our server, we opted to compare results using 295 randomly selected genes within our feasible range. This random subset acts as a feasible proxy to bring insights into the relationship between all other genes and the highly expressed genes in the brain and immune system.

In the remainder of the paper, we will denote by ℛ the set of 295 randomly selected human genes.

### Genes from different families

We selected an additional control gene set that was not based on random sampling, but instead accounted for genes from distinct human gene families.

Two data tables were obtained from HGNC [27]: (1) Human gene families with a typical gene assigned for a subset of families, and (2) a hierarchy of the gene families, where each row represents a mapping between a pair of parent family id and a child family id. We then identified all the human gene families at the top level (i.e., without a parent gene family), among which 382 families as a single assigned typical gene. We then identified all top-level human gene families (i.e., families without a parent), among which 382 families had a single assigned typical gene. This set of 382 genes was used as the control gene set, with each gene representing a distinct human gene family, enabling comparison with brain and immune gene sets from a gene-family perspective.

Ideally, we can process genes genes from both top-level and lower-level gene families. However, due to computational and resource constraints, we limited the analysis to typical genes from top-level families. While this restriction reduces the total number of genes analyzed, it may also reduce bias associated with unbalanced gene family sizes (e.g., large differences in the number of child families per parent family). Out of the 382 genes, 13 genes that are not identified in any species, including human, were excluded from further analysis.

In the remainder of the paper, we will denote by ℱ the set of these 369 human genes from distinct gene families. Genes overlapping with ℬ, ℐ, 𝒞, or ℛ are not removed from ℱ.

### Protein sequences of human and mouse genes

The protein sequences of the above genes were obtained from the ID mapping service on UniProt [34] for both human and mouse genes.

Only the reviewed proteins are used in further analysis. For genes that can be mapped to multiple protein sequences, only the first protein appearing in the UniProt result table was processed.

### Methods

**Fig 1:**
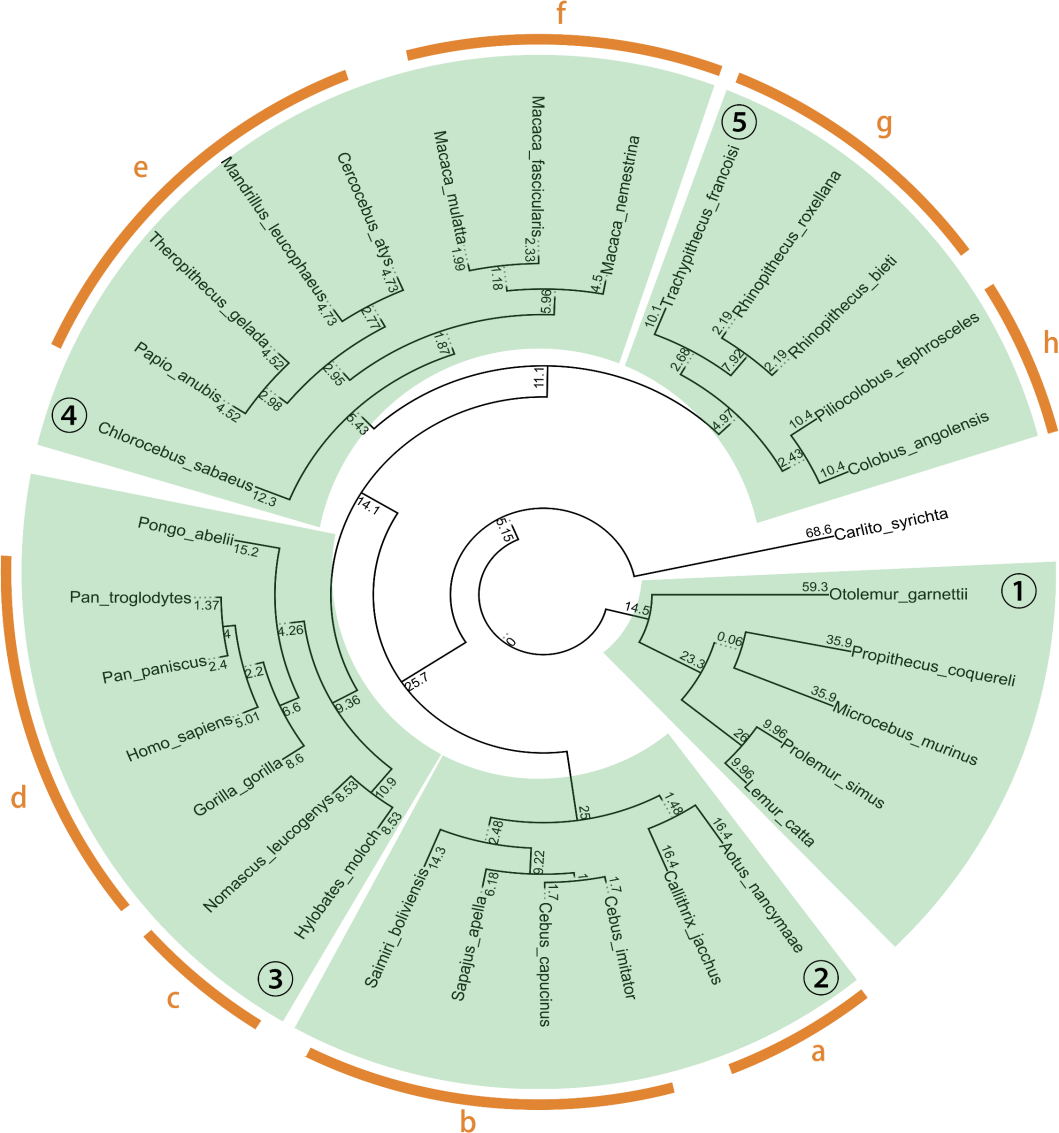
The timetree of the 32 primate species used in this study. The green areas display the partition of the six large clades, while the orange areas further divide them into the eight small clades as described in Section Mapping absent genes to primate clades. The divergence times of the species were obtained from the TimeTree database [38]. Numeric labels represent large clades, while alphabetic labels represent small clades. One species *Carlito syrichta* is not included in any clade as it is far away from other species on the timetree. Human (*Homo sapiens*) is not included in any clade during further analysis processes in human genes, but included in the tree to show the distances from other species to human.

To detect human genes in other species, we employed BLAST to align the protein sequences of these genes with the coding sequences of other species. After applying identity and coverage thresholds to the BLAST results, each gene was assigned to be present or absent in each primate species. Additionally, primate species were divided into groups of large clades and small clades, as illustrated in Fig 1. This study adheres to the widely accepted definition of a clade as a collection of species that are descendants of a common ancestor. A gene absent from all the species from one clade was identified as absent from the entire clade.

### Detecting genes in primate and nonprimate CDS

BLAST [39] was a widely used method for gene presence detection [40–42]. In particular, we use the TBLASTN method that searches a protein query against a database of nucleotides, to search for similar coding sequences in the 32 primate species and 4 non-primate species. The TBLASTN tool was downloaded and locally ran on our server. Nucleotide references were constructed locally from the CDS data of 32 primate species. Searches were conducted for genes in ℬ, ℐ, ℛ, and ℱ using the default parameters provided by BLAST. The resulting data were then filtered to include only those sequences possessing a minimum of 80% similarity in both identity and query coverage per subject.

To reduce the effect of potential bias introduced by thresholds, we have in addition applied a more relaxed threshold of 60% identity and 60% query coverage per subject for BLAST results. Supplementary Materials contain tables generated with this threshold, applied to (1) all species and (2) non-primate species, the latter accounting for their greater evolutionary divergence from human. Notably, results obtained under this relaxed threshold support the same conclusions as those derived from the 80% thresholds.

### Mapping absent genes to primate clades

The 32 primate species were first separated into a list of large clades with sizes between five and eight, then into a list of small clades with sizes between two and three, as depicted in Fig 1. For each large or small clade, each gene was assigned to one of three statuses: present, absent, or unknown. A gene was only assigned to be present in one clade if this gene was observed in all the species in that clade, and absent only if it was not found in any species of the clade. If the data did not clearly support either presence or absence, the gene was classified as unknown. The thresholds of 80% identity and 80% query coverage per subject on BLAST results were applied before the clade assigning process.

### Existence patterns in non-primate species

We encode the existence of genes in the non-primate species as bit strings with bits in the following order: *Mus musculus* (mouse), *Canis lupus familiaris* (dog), *Orycteropus afer afer* (anteater), and *Danio rerio* (zebrafish), where 1 represents presence and 0 represents absence.

In particular, a bit string of 0000 indicates a gene is absent from all the non-primate species examined. A bit string of 1111 indicates the presence of the gene in all the non-primate species. A bit string of 1110 indicates the presence of the gene in all the mammalian non-primate species. A bit string of 1000 indicates the gene is mouse specific across the four non-primate species.

## Results

### Human brain and immune-related genes in non-human species

Table 1 represents the distribution of ℬ, ℐ, 𝒞, and ℛ among 31 non-human primates and 4 non-primate species. We use *N* to represent the total number of genes of a gene set, *J* to represent the total number of genes out from each gene set that were identified in all 31 non-human primates, *K* to represent the total number of genes out from each gene set that were identified in a proper subset of 31 non-human primates, *L* to represent the total number of genes out from each gene set that were identified in none of 4 non-primate species. Then the “primate specific ratio” is defined as $\alpha =\frac{L}{N}$.

**Table 1:** The distribution of ℬ, ℐ, 𝒞, ℛ, and ℱ among 31 non-human primates and 4 non-primate species. We use *N* to represent the total number of genes of a gene set, *J* to represent the total number of genes out from each gene set that were identified in all 31 non-human primates, *K* to represent the total number of genes out from each gene set that were identified in a proper subset of 31 non-human primates, *L* to represent the total number of genes out from each gene set that were identified in none of 4 non-primate species. We call $\alpha =\frac{L}{N}$ the “primate specific ratio”.

Gene Set	Total number of genes *N*	Number of genes identified in all 31 primates *J*	Number of genes identified in a proper subset of the 31 primates *K*	Number of genes identified in none of the 4 non-primate species *L*	Primate specific ratio $\frac{L}{N}$
ℬ	1019	352	667	85	0.08
ℐ	586	192	394	115	0.20
𝒞	245	78	167	27	0.11
ℛ	295	73	222	66	0.22
ℱ	369	134	235	57	0.15

Overall, the genes highly expressed in the brain (ℬ and 𝒞) have lower primate specific ratios, approximately 0.1. This finding indicates that many genes highly expressed in the brain are also present in non-primate species examined in this study, suggesting they are not primate specific. On the contrary, genes highly expressed in the immune system, but not in the brain, have a primate specific ratio similar to the two control gene sets, approximately 0.15 to 0.2. Compared to genes highly expressed in the brain, control genes and and genes highly expressed in the immune system are less found in the examined non-primate species.

To statistically assess our results, we employed Fisher’s exact test [43] to compare the proportion of primate-specific genes in ℬ, ℐ, and 𝒞 with those in ℛ and ℱ, as well as to compare the two control sets with each other. The results presented in Table 2 show that both ℬ and 𝒞 have lower primate-specific ratios than ℛ, and that ℬ also has a lower primate-specific ratio than ℱ. By contrast, no statistically significant differences are detected between ℐ and either ℛ or ℱ, nor between 𝒞 and ℱ. The difference between ℛ and ℱ reaches a weaker significance (*p* < 0.05), suggesting a modest distinction between the gene-family-based control and the randomly sampled control.

**Table 2:** Contingency tables and Fisher’s exact test results for pairwise comparisons. Positive cases are primate-specific genes, while negative cases are genes that are not primate specific. Statistical significance is denoted by asterisks: results with p-values less than 0.01 are indicated by two asterisks (**), whereas results with p-values less than 0.05 are indicated by one asterisk (*). An odds ratio larger than 1 suggests the group is more likely to be primate specific compared to ℛ or ℱ.

Comparison	Group	Positive	Negative	Total	Odds Ratio	Fisher’s p-value
ℬ vs. ℛ	ℬ	85	934	1019	0.316	4.094e-10**
	ℛ	66	229	295		
ℐ vs. ℛ	ℐ	115	471	586	0.847	0.1933
	ℛ	66	229	295		
𝒞 vs. ℛ	𝒞	27	218	245	0.429	0.0003**
	ℛ	66	229	295		
ℬ vs. ℱ	ℬ	85	934	1019	0.498	0.0002**
	ℱ	57	299	369		
ℐ vs. ℱ	ℐ	115	471	586	1.336	0.9584
	ℱ	57	299	369		
𝒞 vs. ℱ	𝒞	27	218	245	0.678	0.07339
	ℱ	57	299	369		
ℱ vs. ℛ	ℱ	57	312	369	0.634	0.01474*
	ℛ	66	229	295		

By observing the significant results, gene in 𝒞 are more similar to ℬ than ℐ in terms of odd ratios and p-values. It is possible that genes highly expressed in both brain and immune tissues are more likely to have originated similarly to brain genes, which emerged earlier than immune-related genes in general. One potential interpretation is that these genes are more likely to be initially highly expressed in the brain before acquiring high expression in immune tissues, rather than vice versa.

**Fig 2:**
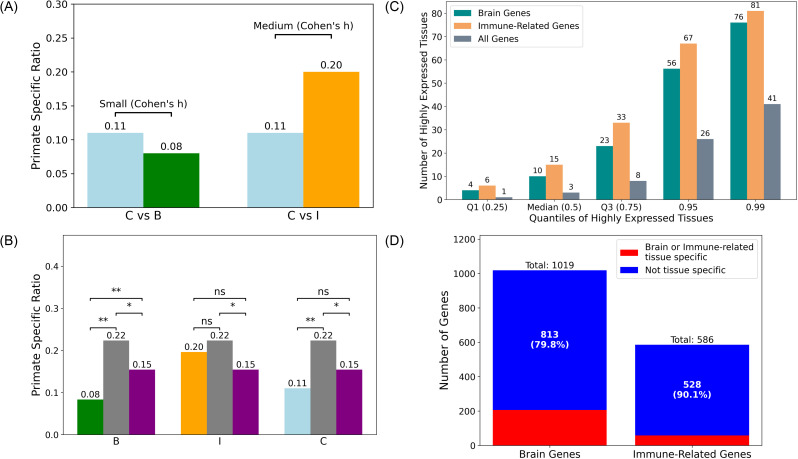
Statistical comparisons and gene distribution summary. **(A)** Cohen’s h values for pairwise comparisons between 𝒞 and the other two groups: ℬ and ℐ. Primate Specific Ratios are calculated as in Table 1. A higher absolute Cohen’s h indicates a greater difference in ratios between the two compared groups. Green bar represents ℬ, orange bar represents ℐ, and blue bars represent 𝒞. **(B)** Results of Fisher’s exact tests for pairwise comparisons between each of ℬ, ℐ, and 𝒞 and each control group (ℱ and ℛ), together with the comparison between ℱ and ℛ. Primate Specific Ratios are calculated as in Table 1. Statistical significance is denoted by asterisks. Results with p-values less than 0.01 are indicated by two asterisks (**), results with p-values less than 0.05 are indicated by one asterisk (*), and all other results are marked as *ns*. Green bar represents ℬ, orange bar represents ℐ, blue bar represents 𝒞, gray bars represent ℛ, and purple bars represent ℱ. **(C)** The number of tissues corresponding to each quantile for the brain genes and immune-related genes. Green bars represent ℬ, orange bars represent ℐ, and gray bars represent all genes that have at least one highly expressed tissue in Human Protein Atlas [32,33]. **(D)** The number and ratio of genes highly expressed specifically in brain tissues or immune-related tissues, out of 1019 brain genes and 586 immune-related genes, respectively.

To further assess whether 𝒞’s primate-specific proportion more closely resembles that of ℬ or ℐ, we apply Cohen’s h [44], a standardized measure of effect size for differences between proportions. Table 3 presents the results. This result also indicates that the genes highly expressed in both the brain and immune system is more similar in their evolutionary origin to brain genes rather than immune-related genes. To better visualize our results, the Fisher’s exact test and‌‌ Cohen’s h comparing different groups are also depicted in Fig 2A and Fig 2B.

**Table 3:** Proportion of positive or negative outcomes for each group, and Cohen’s h values for pairwise comparisons between 𝒞 and the other two groups: ℬ and ℐ. Here, positive cases are primate-specific genes, while negative cases are genes that are not primate specific. A higher absolute Cohen’s h indicates a greater difference in proportions.

Group	Positive (%)	Total n
ℬ	8	1019
ℐ	20	586
𝒞	11	245
**Comparison**	**Cohen’s h**	**Interpretation**
𝒞 vs ℬ	0.091	Small
𝒞 vs ℐ	−0.241	Medium

Among the 751 genes found in all the 31 non-human primate species from the five gene sets, 748 were also present in at least one of the four non-primate species examined. This observation supports the intuition that genes shared among most of the primates are more likely to trace back to common ancestors predating the origin of primates. The three exceptions that are absent from all the four non-primate species are gene *LIPA* from ℬ, gene *EXTL1* from 𝒞, and gene *TDRD1* from ℱ. Furthermore, given that 323 genes in total were identified as absent from all four non-primate species, it is unlikely that the observed pattern simply results from a generally low rate of gene absence in non-primates. Even if we limit the non-primate species to the three well studied non-primates (i.e., mouse, dog, and zebrafish), the numbers remain the same, as none of these genes are found exclusively in anteaters and absent from all three others.

### Widely expressed and tissue-specific genes

In the above results, a confounding factor is how widely these genes are expressed. Could the observed results for brain genes simply reflect that genes highly expressed in the brain tend also to be widely expressed across other tissues? To investigate this confounding factor and strengthen the robustness of our controls, we examined the breadth of tissues in which brain and immune-related genes are highly expressed, and whether focusing specifically on widely expressed genes yields different conclusions.

We first investigated how broadly are brain and immune-related genes expressed in the original data obtained from Human Protein Atlas [32,33]. As described in Section Methods, a gene is classified as highly expressed in a tissue if the gene–tissue pair has both a “High” expression level and “Enhanced” reliability score in the original data. Fig 2C shows the number of tissues at each quantile for the 1019 brain genes and 586 immune-related genes. Based on the quantile distribution, we chose the median value for brain genes, 10, as the threshold for widespread expression in this study. Genes expressed in 10 or more tissues are classified as widely expressed.

We then assessed whether the difference between brain genes (ℬ) and immune-related genes (ℐ) is primarily driven by widely expressed genes. Contingency tables for primate-specific genes in each group, along with the results of Fisher’s exact tests, are presented in the first two rows of Table 4. The results indicate that, compared to genes widely expressed in at least 10 tissues, genes with narrower high expression profiles are more likely to contribute to differences in primate specific ratios between brain and immune-related genes.

**Table 4:** Contingency tables and Fisher’s exact test results for three comparisons: (1) the subsets of “wide 10” genes (genes highly expressed in at least 10 tissues) in ℬ and ℐ, (2) the subsets of “narrow 10” genes (genes highly expressed in less than 10 tissues) in ℬ and ℐ, and (3) the subsets of “tissue specific” genes, the genes highly expressed exclusively in brain tissues in ℬ and genes highly expressed exclusively in immune-related tissues in ℐ. Positive cases are primate-specific genes, while negative cases are genes that are not primate specific. A p-value less than 0.01 are marked with two asterisks (**), indicating a more statistically significant difference. An odds ratio less than 1 suggests the group in ℬ is less likely to be primate specific compared to the group in 𝒞. The union of “wide 10” and “narrow 10” genes corresponds to the complete gene sets (1019 and 586) reported in Table 2, where results for the full sets are shown. This separation allows assessment of whether statistical significance observed in the full gene sets is driven primarily by the “wide 10,” “narrow 10”, or “tissue specific” subset.

Comparison	Group	Positive	Negative	Total	Odds Ratio	Fisher’s p-value
Wide 10	ℬ	39	205	244	0.849	0.293
	ℐ	41	183	224		
Narrow 10	ℬ	45	730	775	0.240	5.927e-13**
	ℐ	74	288	362		
Tissue Specific	ℬ	14	192	206	0.128	1.317e-07**
	ℐ	21	37	58		

To further validate our findings, we also analyzed genes that are highly expressed specifically in brain or immune-related tissues, instead of only narrowly expressed in less than 10 tissues. Fig 2D illustrates the number and proportion of genes with high expression specific to brain or immune-related tissues. We then examined whether the observed differences in primate-specific ratios between brain genes (ℬ) and immune-related genes (ℐ) can be driven by these tissue-specific genes. The results shown in the third row of Table 4 indicate that these genes are indeed likely to have contributed to the differences.

Together, these analyzes suggest that the differences in primate-specific ratios between brain and immune-related genes (as shown in Table 2) are unlikely driven by the genes with broad expression, but the genes more narrowly expressed or uniquely expressed in brain or immune-related tissues.

### Genes absent from entire primate clades

#### Overview.

Here, we investigate more closely the genes that are absent from certain primate clades, rather than from the entire set of primate or non-primate species. Out of the genes exist in at least one of the 31 primate species, we examine genes absent from both large and small clades, as defined in Fig 1. Overall, we found that among genes in ℬ, 56 are missing from large clades and 58 from small clades. For genes in ℐ, 72 are absent from large clades and 49 from small clades. For genes in ℛ, 54 are absent from large clades and 41 from small clades. For genes in ℱ, 47 and 31 genes are missing from large and small clades, respectively.

Table 5 presents a more detailed summary of gene absences across primate clades. We use *N* to represent the total number of genes of a gene set, *P* to represent the total number of genes that are absent from large clades, and *Q* to represent the total number of genes that are absent from small clades. Then the “large clade absent ratio” and “small clade absent ratio” are defined as $\beta =\frac{P}{N}$ and $\gamma =\frac{Q}{N}$, respectively.

**Table 5:** The absence of genes from ℬ, ℐ, 𝒞, ℛ, and ℱ in primate clades within 31 non-human primates, where the large and small clades are defined in Fig 1. Within the genes exist in at least one of the 31 primate species, we use *N* to represent the total number of genes of a gene set, *P* to represent the total number of genes that are absent from large clades, and *Q* to represent the total number of genes that are absent from small clades. We call $\beta =\frac{P}{N}$ and $\gamma =\frac{Q}{N}$ “large clade absent ratio” and “small clade absent ratio” respectively.

Gene Set	Total number of genes *N*	Number of genes absent from large clades *P*	Number of genes absent from small clades *Q*	Large clade absent ratio $\frac{P}{N}$	Small clade absent ratio $\frac{Q}{N}$
ℬ	1019	56	58	0.05	0.06
ℐ	586	72	49	0.12	0.08
𝒞	245	14	16	0.06	0.07
ℛ	295	54	41	0.18	0.14
ℱ	369	47	31	0.13	0.08

To statistically assess the ratios shown in Table 5, we use Fisher’s exact test [43] to compare the proportion of positive outcomes (i.e., the genes absent from large clades or small clades) in ℬ, ℐ, and 𝒞 to that of ℛ or ℱ, as well as a comparison in between the two cotrol sets ℛ and ℱ. The Fisher’s exact test results are summarized in Table 6.

**Table 6:** Contingency tables and Fisher’s exact test results for pairwise comparisons. Positive cases genes absent from large clades. Statistical significance is denoted by asterisks: results with p-values less than 0.01 are indicated by two asterisks (**), whereas results with p-values less than 0.05 are indicated by one asterisk (*). An odds ratio larger than 1 suggests the group is more likely to be absent from large clades compared to ℛ or ℱ.

Comparison	Group	Positive	Negative	Total	Odds Ratio	Fisher’s p-value
ℬ vs. ℛ	ℬ	56	963	1019	0.260	1.041e-10**
	ℛ	54	241	295		
ℐ vs. ℛ	ℐ	72	514	586	0.625	0.0114*
	ℛ	54	241	295		
𝒞 vs. ℛ	𝒞	14	231	245	0.270	5.462e-06**
	ℛ	54	241	295		
ℬ vs. ℱ	ℬ	56	963	1019	0.398	1.171e-05**
	ℱ	47	322	369		
ℐ vs. ℱ	ℐ	72	514	586	0.960	0.4561
	ℱ	47	322	369		
𝒞 vs. ℱ	𝒞	14	231	245	0.415	0.0026**
	ℱ	47	322	369		
ℱ vs. ℛ	ℱ	47	322	369	0.651	0.0306*
	ℛ	54	241	295		

The analysis shows that, in both the large and small primate clades, genes highly expressed in the brain or immune system (ℬ, ℐ, and 𝒞) tend to have lower clade-absent ratios compared to randomly selected human genes (ℛ) and human genes from distinct gene families (ℱ). Notably, when comparing genes that are absent from the entire set of primate species with those present in some but absent in certain clades, immune-related genes (ℐ), despite being similar to random genes (ℛ) in their overall primate-specific ratios, show a more structured pattern of clade-specific absence, as detailed later and summarized in Table 8. This implies a more organized evolutionary loss or divergence of immune-related genes. Further study of the primates in these clades may help clarify the factors involved in this pattern and deepen our understanding of human gene evolution.

For all the genes absent from large or small primate clades, the majority of their bit strings is 0000. This indicates that they are also absent in the 4 non-primate species and likely to have an origin later than primate origin. In more detail: (1) In ℬ, 41 out of 55 genes that are absent from large clades have this 0000 bit string. Of the 58 genes absent from small clades, 24 genes have the 0000 bit string. This is the only case where 0000 is not the most frequent bit string. (2) In ℐ, 68 out of 72 genes absent from large clades and 38 out of 49 genes absent from small clades have the 0000 bit string. (3) For genes in ℛ, 32 out of 41 for large clades and 49 out of 54 for small clades. (4) For genes in ℱ, 45 out of 47 for large clades and 23 out of 31 for small clades.

#### Genes absent from large clades.

We have further examined the other tissues these genes are highly expressed in with an “Enhanced” level evidence. Table 7 shows the genes in ℬ that are absent from at least two large clades and Table 8 shows genes in ℐ that are absent from at least three large clades. It also provides information on the number of tissues where these genes are highly expressed, the clades they are absent from, and their bit strings.

**Table 7:** Expression and existence bit strings of selected genes. Selected genes are those in set ℬ that do not belong to set 𝒞 (denoted ℬ⧵𝒞), and that are absent from at least two large clades. For genes that are highly expressed in no more than five tissues, the tissues are listed out.

Gene	Gene set	Number of highly expressed tissues	List of highly expressed tissues	Number of absent large clades	bit string
*ASCL1*	ℬ	3	Hippocampus, cerebral cortex, cerebellum	All (except *Homo sapiens*)	0000
*MT-ND3*	ℬ	16		4 (Clade 1, 2, 4, 5)	0000
*SP100*	ℬ	15		2 (Clade 1, 2)	0000
*AHNAK2*	ℬ	11		2 (Clade 1, 2)	0000
*PRR18*	ℬ	1	Hhippocampus	2 (Clade 1, 2)	0000
*CCSAP*	ℬ	4	Hippocampus, caudate, cerebral cortex, cerebellum	2 (Clade 1, 2)	0000
*MADCAM1*	ℬ	5	Cerebellum, small intestine, rectum, colon, duodenum	2 (Clade 1, 5)	0000
*PODXL*	ℬ	4	Cerebral cortex, placenta, fallopian tube, kidney	2 (Clade 1, 4)	0000
*NLRP1*	ℬ	3	Hippocampus, cerebral cortex, skin 1	2 (Clade 1, 2)	0000
*CMTM5*	ℬ	2	Cerebellum, soft tissue 2	2 (Clade 1, 2)	0000
*C9orf50*	ℬ	2	Cerebral cortex, testis	2 (Clade 1, 2)	0000
*IQCN*	ℬ	2	Cerebral cortex, testis	2 (Clade 1, 2)	0000
*MT3*	ℬ	4	Hippocampus, caudate, cerebral cortex, cerebellum	2 (Clade 4, 5)	0100
*SYN1*	ℬ	1	Cerebellum	2 (Clade 1, 2)	0010

^1^Skin 1 contains skin samples that have been exposed to the sun [32,33].

^2^Expressions in soft tissue 2 are obtained from a second set of samples, different from the set included in soft tissue 1, to include as many cell types in soft tissues as possible [32,33].

**Table 8:** Expression and existence bit strings of selected genes. Selected genes are genes in ℐ∪𝒞 that are absent from at least two large clades. For genes that are highly expressed in no more than five tissues, the tissues are listed out.

Gene	Gene set	Number of highly expressed tissues	List of highly expressed tissues	Number of absent large clades	bit string
*TGOLN2*	𝒞	40		2 (Clade 1, 2)	0000
*SCAF4*	𝒞	21		2 (Clade 1, 2)	0000
*MT-CO2*	𝒞	44		2 (Clade 1, 2)	0000
*BST2*	ℐ	10		4 (Clade 1, 2, 4, 5)	0000
*DEF6*	ℐ	7		3 (Clade 1, 2, 5)	0000
*GBP4*	ℐ	8		2 (Clade 1, 5)	0000
*MNDA*	ℐ	7		2 (Clade 1, 2)	0000
*GIMAP4*	ℐ	5	Lymph node, tonsil, adrenal gland, placenta, skin 21	2 (Clade 1, 2)	0000
*LAIR1*	ℐ	4	Bone marrow, lymph node, placenta, appendix	2 (Clade 1, 2)	0000
*MCEMP1*	ℐ	3	Lung, bone marrow, spleen	2 (Clade 1, 2)	0000
*CD1A*	ℐ	3	Thymus, skin 1, skin 2	2 (Clade 1, 2)	0000
*CLEC12A*	ℐ	2	Bone marrow, spleen	2 (Clade 1, 2)	0000
*S100A9*	ℐ	10		2 (Clade 1, 2)	0000
*CD177*	ℐ	6		2 (Clade 1, 2)	0000
*TCL1A*	ℐ	2	Lymph node, appendix	2 (Clade 1, 2)	0000
*DEFA1*	ℐ	2	Bone marrow, spleen	2 (Clade 1, 2)	0000
*ELANE*	ℐ	1	Bone marrow	2 (Clade 1, 2)	0000
*FCN1*	ℐ	2	Bone marrow, placenta	2 (Clade 1, 2)	0000
*S100A12*	ℐ	2	Bone marrow, spleen	2 (Clade 1, 2)	0000
*CTSG*	ℐ	1	Bone marrow	2 (Clade 1, 2)	0000
*PTPRCAP*	ℐ	8		2 (Clade 1, 2)	0000

^1^Skin 2 contains skin samples from areas that are not exposed to the sun [32,33].

Among 55 brain genes absent from large clades, there are 5 genes particularly absent from clade 2. The remaining 50 genes are all absent from clade 1, with 14 additionally absent from clade 2. Then there are 4 genes absent particularly from clade 2, and 1 gene absent from clade 4 and 5.*ASCL1* is specific to human as it is absent from all the primate clades excluding the human species. Among 72 immune-related genes absent from large clades, there are 3 genes particularly absent from clade 2. The other 69 genes are all absent from clade 1, with 1 gene *GBP4* additionally absent from clade 5 and 20 genes additionally absent from clade 2. Out from the 20 genes, *BST2* is also absent from clade 4 and 5, *DEF6* is also absent from clade 5.

As mentioned in Section Existence patterns in non-primate species, the bit string 0000 indicates that the genes are absent from all the 4 examined non-primate species. Accordingly, most of the genes displayed in Table 7 are not found in any of the non-primate species, with two exceptions *MT3* found in dog and *SYN1* found in anteater.

For genes in 𝒞 that are absent from at least two large clades, the numbers of their highly expressed tissues are all larger than 20, with the mean and median values within 𝒞 being 24.29 and 24.5, respectively. For genes in ℬ⧵𝒞 that are absent from at least two large clades, they are either highly expressed in more than 10 tissues, or highly expressed in no more than two systems including brain (see Section Newly emerged brain and immune-related genes in primates for more details), with an only exception *PODXL*. For genes in ℐ⧵𝒞 that are absent from at least two large clades, all the genes are expressed in no more than 10 tissues. Of these genes, most are expressed in 5 or fewer tissues and are predominantly associated with the immune system, although some of the tissues may also function within other biological systems.

#### Genes absent from small clades.

Table 9 details the notable genes that are absent from small primate clades. Among immune-related genes and brain genes absent from small clades, all 5 immune-related genes with an 1110 or 1111 bit string are notably expressed in more than 15 tissues each.

**Table 9:** Expression and existence bit strings of selected genes. Selected genes are genes that are absent from small clades and possess bit strings of 1111 or 1110, within ℬ, ℐ, and 𝒞. No genes from ℐ satisfy this condition. For genes that are highly expressed in less than five tissues, the tissues are listed out.

Gene	Gene set	Bit string	Number of highly expressed tissues	List of highly expressed tissues	Small clades absent from
*MT-CO1*	𝒞	1111	31		Clade a, b, c, d
*PPWD1*	ℬ	1111	22		Clade a, d, f, h
*FLOT1*	𝒞	1111	18		Clade h
*BHLHE22*	ℬ	1111	1	Cerebellum	Clades a, d
*KCNA2*	ℬ	1111	1	Cerebral cortex	Clade a, h
*CASKIN1*	ℬ	1111	1	Cerebellum	Clade h
*ISOC2*	𝒞	1110	40		Clade c
*RAD23B*	ℬ	1110	39		Clade a, b
*GPATCH11*	𝒞	1110	36		Clade c
*GLOD4*	𝒞	1110	29		Clade h
*MAOA*	ℬ	1110	27		Clade g
*JUN*	ℬ	1110	9		Clade a, d
*SHANK3*	ℬ	1110	5	Hippocampus, caudate, cerebellum, endometrium, kidney	Clade h
*KCNQ2*	ℬ	1110	3	Caudate, hippocampus, testis	Clade c
*P2RY12*	ℬ	1110	2	Cerebellum, placenta	Clade d
*APP*	ℬ	1110	1	Cerebellum	Clade a
*ITPKA*	ℬ	1110	1	Cerebellum	Clade h
*DLG2*	ℬ	1110	1	Cerebellum	Clade h

Note that for brain genes absent from small clades, there are in total 10 genes specifically highly expressed in a single tissue, among which 8 genes are highly expressed exclusively in the cerebellum. Five out of these eight exhibit a non-primate bit string of either 1111 or 1110 as shown in Table 9. The remaining three genes expressed exclusively in the cerebellum are found in at least one non-primate species, displaying non-primate bit strings 0110, 0010, and 1000, respectively.

### Validation using subsampling and consensus high expression gene sets

It is notable that, compared with immune-related tissues, brain tissues have a larger number of highly expressed genes. This is consistent with findings from tissue-expression studies showing that the brain has a particularly large number of tissue-enriched genes relative to most other human tissues [45,46]. To assess whether the larger size of ℬ influenced the results, we performed a resampling analysis in which subsets of ℬ were repeatedly sampled 1000 times without replacement to match the size of ℐ. The results are reported in Supplementary Tables S9 and S10 in S1 File, and support the main conclusions by showing that the observed patterns are not explained solely by the larger size of ℬ.

To assess whether our main findings depend on the use of a single, categorically defined resource, we repeated the analysis using the overlap of genes identified as highly expressed in brain and immune-related tissues compared to an additional quantitative proteomics databases: PaxDB v6.0 [47]. As the two resources differ in sample composition and underlying methodology, exact agreement across databases is not expected [48]. Our aim was therefore not strict concordance, but to test whether similar results can be achieved with genes that are consistently classified as highly expressed across all three resources.

For brain, direct tissue-level matching across databases was constrained by differences in coverage and in the samples contributing to each resource. We therefore used the broader “brain” entries available in PaxDB as the closest available basis for cross-database comparison. In PaxDB, the broader brain entry is derived from source datasets distinct from the tissue-level brain samples [47]. For immune-related tissues, because PaxDB do not provide an “immune” category comparable to “brain”, we used data from bone marrow and lymph node, which overlap with two of the three immune tissues used in Human Protein Atlas. Thymus was not available in the database. Accordingly, the cross-database comparison was based on the most comparable data sets available, although the underlying tissue composition was not fully aligned across resources.

To enable the comparison across databases, PaxDB abundance values were converted into approximate low, medium, and high expression categories using within-tissue percentile ranks: low (< 25^th^), medium ($\geq 25^{\text{th}}$ and < 75^th^), and high ($\geq 75^{\text{th}}$ percentile). This differs from the Human Protein Atlas [32,33] tissue-expression scoring system, which combines staining intensity with the fraction of stained cells and further refined by expert annotation [49], but produces a comparable classification for cross-database overlap.

We then retained only genes classified as high in both resources, Human Protein Atlas and PaxDB, and repeated the analyses. This overlap reduced the number of brain genes to 536, immune genes to 256, and genes highly expressed in both brain and immune tissues to 74. We then use ${ℬ}^{*}$ and ${ℐ}^{*}$ to denote the consensus sets of genes highly expressed in brain-related and immune-related tissues, respectively, between Human Protein Atlas and PaxDB. We define ${𝒞}^{*}={ℬ}^{*}\cap {ℐ}^{*}$.

The results of these analyses are summarized in Table 10 and Table 11, and the corresponding Cohen’s h and Fisher’s exact test results are provided in Supplementary Table S11, S12, and S13 in S1 File. With the constraint of limited numbers in certain categories (e.g., number of genes absent from large clades in ${𝒞}^{*}$), the overall pattern remained consistent with the analyses based on Human Protein Atlas high-expression genes alone, indicating that the main conclusions do not rely on Human Protein Atlas as a single data source.

**Table 10:** The distribution of ${ℬ}^{*}$, ${ℐ}^{*}$, ${𝒞}^{*}$, ℛ, and ℱ among 31 non-human primates and 4 non-primate species. We use *N* to denote the total number of genes in a gene set, *J* the number identified in all 31 non-human primates, *K* the number identified in a proper subset of the 31 non-human primates, and *L* the number identified in none of the 4 non-primate species. We define $\alpha =\frac{L}{N}$ as the primate-specific ratio.

Gene Set	Total number of genes *N*	Number of genes identified in all 31 primates *J*	Number of genes identified in a proper subset of the 31 primates *K*	Number of genes identified in none of the 4 non-primate species *L*	Primate specific ratio $\frac{L}{N}$
${ℬ}^{*}$	536	208	328	25	0.05
${ℐ}^{*}$	256	84	172	52	0.20
${𝒞}^{*}$	74	27	47	4	0.05
ℛ	295	73	222	66	0.22
ℱ	369	134	235	57	0.15

**Table 11:** The absence of genes from ${ℬ}^{*}$, ${ℐ}^{*}$, ${𝒞}^{*}$, ℛ, and ℱ in primate clades within 31 non-human primates, where the large and small clades are defined in Fig 1. Within the genes exist in at least one of the 31 primate species, we use *N* to represent the total number of genes of a gene set, *P* to represent the total number of genes that are absent from large clades, and *Q* to represent the total number of genes that are absent from small clades. We call $\beta =\frac{P}{N}$ and $\gamma =\frac{Q}{N}$ “large clade absent ratio” and “small clade absent ratio” respectively.

Gene Set	Total number of genes *N*	Number of genes absent from large clades *P*	Number of genes absent from small clades *Q*	Large clade absent ratio $\frac{P}{N}$	Small clade absent ratio $\frac{Q}{N}$
${ℬ}^{*}$	536	22	17	0.04	0.03
${ℐ}^{*}$	256	38	2	0.15	0.01
${𝒞}^{*}$	74	3	1	0.04	0.01
ℛ	295	54	41	0.18	0.14
ℱ	369	47	31	0.13	0.08

## Discussion

### The early emergence and long preservation of human brain genes

The human genome contains genes of various origins, emerging at different time points in evolution history. In this study, one question we are interested in is whether certain genes, particularly those expressed in different tissues, originated within or predate the primates.

Our results in Section Human brain and immune-related genes in non-human species show that compared to control and immune system genes, a larger proportion of human brain genes are identified in non-primate species. Additionally, results in Section Genes absent from entire primate clades show that fewer brain genes are absent from primate clades than control and immune system genes, especially the large clades. These findings suggest that brain genes have an earlier origin than immune system and control genes, likely predating primates, and have been preserved across primate species. On the other hand, genes highly expressed in immune system have a similar distribution to control human genes in both large primate clades and non-primate species. Genes that are highly expressed in both brain and immune tissues are more likely to have originated like brain genes, with initial high expression in the brain before acquiring high expression in immune tissues, rather than the reverse.

Previous work has shown that primate and human specific genes can be associated with certain tissues, including the human-specific genes *ARHGAP11B* and *NOTCH2NL*, which are associated with neocortical expansion [10–14]. Bitar et al. [18] have found involvement of human specific genes in adaptations affecting the immune system, along with brain and metabolism. In addition, although not investigated in this study, primate and human specific genes are also associated with diseases such as cancer [8], primary microcephaly [17], infantile cardiomyopathy and Kearns–Sayre syndrome [15], there are species specific genes reported to have associations with reproduction [50].

Previous studies have also shown that the transcript expression levels of many species specific genes, such as primate and human specific genes, are generally low [16,21,51,52] and tissue-specific [21,52] or chromatin region-specific [53], although the expressions at transcript level and protein level are not always linear [54]. Nevertheless, the highly expressed brain genes studied in this study have been generally identified as emerging earlier than primates, which aligns with previous results [55], with certain exceptions that will be discussed in Section Newly emerged brain and immune-related genes in primates.

In our results, many of the highly expressed genes in the brain are shown not to be primate or human specific. However, the distinction between humans and other species, including primates, may not hinge on a large number of highly expressed genes. It is possible that genes with low expressions, as suggested in previous study [10–14], play a significant role differentiating humans and primates from other species. Additionally, the results in this study indicate that although brain, immune-related, and reproduction-related genes are all reported to be associated with primate and human specific genes, their evolution rates can be different. For immune and reproduction genes, they have been demonstrated to have evolved rapidly [28,29], aligned with our results, and possibly have been specific to primate and human by a certain proportion of their kind of genes. Brain genes, in contrast, do not evolve quickly, but can be specific to human and primates with only a relatively small number of genes making critical differences [26].

### Newly emerged brain and immune-related genes in primates

For genes absent from entire primate clades, the question arises as to whether these genes were eliminated from species within the clade or emerged in other species. This cannot be definitively answered without further experimental data. In this study, we attempted to approximate an answer by examining their sequence presence in non-primate species.

The results in Section Genes absent from entire primate clades demonstrate that for genes absent in primate clades, the predominant bit string is 0000. Thus, we infer that these genes are less likely to have been eliminated from an entire clade. Rather, they may have newly emerged within primates, particularly after the divergence of the common ancestors of the primate and non-primate species examined in this study.

Proportionally, genes highly expressed in the brain and immune system tend to be absent in fewer primate clades compared to a selection of control human genes, as indicated in Table 5. This suggests that genes highly expressed in the brain, or in both the brain and immune systems, have exhibited a lower emergence rate in primates compared with control genes. This trend could point to unique evolutionary processes influencing the development of brain and immune-related genes in primates. An in-depth exploration of the primate species where these genes are absent could offer valuable insights into why these genes are uniquely absent and present in certain primate clades, including humans.

It is noteworthy that, while the majority of genes with high expression in the brain have possibly emerged predating the primates, there are several exceptions. One such exception is the gene *LIPA*, which is found in all 32 primate species but absent in the 4 non-primate species examined. Section Genes absent from entire primate clades shows both brain and immune-related genes that are absent from large primate clades, with some significant genes absent from multiple large clades as listed in Table 7 and Table 8. This list includes a gene identified as specific to human, *ASCL1*. Among these genes, *ASCL1* is likely to have emerged in human, *LIPA* has probably emerged in the common ancestor of the 32 primates including human, and the other genes have probably emerged in a common ancestor of certain primate clades, thereby not being present in others. The absence of several genes that are highly expressed in various tissues and absent from certain primate clades, may reflect fundamental evolutionary differences among primate species.

We have identified a set of genes in ℬ⧵𝒞 that are absent from no less than two large clades and expressed in no more than five tissues. Among these genes, five of them, including the human specific gene *ASCL1*, are expressed in brain-related tissues only. Most of the other genes are highly expressed specifically in brain and tissues from another organ system. *MADCAM1* is expressed in brain and digestion related tissues. *IQCN* and *C9orf50* are expressed in brain and male reproduction related tissues. *NLRP1* is expressed in brain and skin 1, the skin samples that have been exposed to the sun. *CMTM5* is expressed in brain and soft tissue. An exception, *PODXL* is expressed in three systems, including brain, urine, and female reproduction related tissues. The other three exceptions are expressed in more than 10 tissues. For genes in ℐ⧵𝒞 (genes in set ℐ that do not belong to set 𝒞) that are absent from at least two large clades, most of these genes are expressed in five or fewer tissues and are mainly related to the immune system, though a few tissues might also participate in other organ systems. Further studies on (1) the interactions at genetic level between brain and other systems and (2) the genes specific to immune systems, with a focus on their absence in some primate clades, may present an interesting topic for future research endeavors.

Most of the genes absent from large clades are absent from clade 1, which includes five species from the *Strepsirrhini* order. Many are also absent from clade 2, which includes six species from the *Platyrrhini* order, also known as New World monkeys. The divergence times of *Strepsirrhini* and *Platyrrhini* from other species can be pivotal for further evolutionary studies, indicating critical periods for brain and immune-related gene divergence of primates.

Previous studies have examined the differences between the primates of the *Strepsirrhini* and *Platyrrhini* clades and those of other primate groups, from brain and genetic perspectives. Prior research has indicated that species within *Strepsirrhini* possess smaller average genome sizes compared to other primates [26]. Moreover, these species generally have lower brain volumes [26] and brain-to-body ratios [23]. Distinct from primates more closely related to humans, species in *Strepsirrhini* differ markedly in various aspects, such as diet [56] and nocturnal habit [26]. Studies on the brains of New World monkeys have been conducted in various directions [57–59] and have been revealed to have less significantly folded cerebral cortex than Old World monkeys [59]. Exploring the genetic variations that account for the diverse traits among primates, including human, presents a valuable area for further investigation.

There is not much previous work on the difference of immune systems for different species, especially on *Strepsirrhini* and *Platyrrhini*. With the recent advancements in studies in epidemiology and COVID-19, related works on identifying potential hosts among non-human primates are conducted on primate species closely related to human [60,61]. The further exploration of the differences in immune systems, not only on the genes shared among various species but also those absent from certain clades, can offer valuable insights on human disease and potential hosts of infectious diseases.

### Lost brain and immune-related genes in primate clades

In Section Genes absent from entire primate clades, we report that most of the genes absent in some primate clades but present in mouse, dog, anteater, and partially in zebrafish, are either expressed in over 15 different tissues including those of the immune system or are specifically expressed in the cerebellum or cerebral cortex. This suggests a link between the evolutionary origins of these genes and their functional roles.

Typically, genes expressed in a wide range of tissues are classified as housekeeping genes, essential for the basic functions of life forms not limited to human [62]. Some of these genes demonstrate evolutionary resilience [63]. Recent research has also highlighted the importance of broadly expressed genes in disease progression [64,65]. From our results, the genes expressed widely in multiple tissues could be essential and evolutionarily resilient across most primate species. Their involvement in the immune system and absence in certain primate clades can offer further insights into primate evolution and unique aspects of human immune processes.

Earlier work has aligned mammalian evolution with an expansion of the neocortex and an increase in cerebellar neuron numbers [66,67]. A recent study, comparing human cerebellar genes with those of two eutherians (human and mouse) and a marsupial (opossum, Monodelphis domestica), suggests that many human cerebellar genes have been possibly preserved for more than 160 million years. Our investigation in 32 primates and 4 non-primate species aligns with these findings, indicating that many cerebellum specific genes may have originated before the last common ancestor of the examined species, only being absent from certain primate clades.

While the cerebellum is traditionally known for its role in motor control and coordination [68], recent studies have expanded the understanding of its functions, including cognitive processes and emotion regulation [69–71]. The absence of genes from certain primate clades, notably *MT-CO1*, *BHLHE22*, *ISOC2*, *GPATCH11*, *JUN*, *KCNQ2*, and *P2RY12*, in closely related clades (clade c and d) to human, may be associated with unique cerebellar functions in primates including human. Examples include motor control, cognition, and emotion processes. These findings can reveal insights into further exploration to understand the unique characteristics of human cerebellar genes. It is also notable that the cerebellum is demonstrated to gain neurons more slowly than the cerebral cortex, which is another direction for future investigation [59].

It would be intriguing to investigate further the genes that are specifically expressed in one or two tissues and have been absent from certain primate clades. Regarding genes highly expressed in brain, we have identified *APP*, *DLG2*, *CASKIN1*, *BHLHE22*, *ITPKA*, and *CACNG8* as being specifically expressed in the cerebellum, with *KCNA2* specifically expressed in the cerebral cortex. Conducting more experiments on these individual genes can be insightful.

### Limitations and future work

It is notable that this study is conducted on proteomics data and focuses on primary organ tissues related to the brain or immune system. While proteomics data potentially provide information on biological activity and functional engagement of genes in biological processes, transcriptomic data can be more informative for understanding gene sequences, and their biases can be better corrected using large cohorts. When considering broader tissues related to the brain or immune system, the results reported in this study may not apply.

For genes from distinct gene families, we used the HGNC identification of a typical member gene for each gene group, as well as the gene family hierarchy. This classification depends on HGNC definitions and may not be consistent with alternative approaches to gene family categorization or hierarchy construction.

It is intriguing that the random gene set shows weak but statistically significant differences in some results, which may motivate future work investigating factors associated with gene characteristics, such as the emergence time of genes, in different gene families.

Our knowledge of species examined in this study, excluding humans and established model organisms, remains less comprehensive in comparison. While previous discussions have outlined potential directions for future investigation, in-depth research still relies on more thorough grasp of these species. From a broader perspective, further distinguishing between species closely related to humans and those more distantly related, or associating the gene preservation with the different evolutionary distances from human as quantitative values, may offer valuable insights into human genetics and evolution. With the increasing availability of protein expression data for a broader range of non-human primate species, it would also be interesting to examine their protein expression levels alongside gene sequence preservation to better understand how genes participate in biological processes across different species.

This study also raises another question: in this research, we examined the presence of human genes in other species, but what about the presence of genes from other species in humans? Can the presence of mouse genes in primates and humans provide us with some insights? Will this offer some implications for the recapitulate on how mouse models recapitulate human diseases in medical research? These are all interesting future work questions.

## Conclusion

This study investigates the existence and distribution patterns of human genes with high expression in the brain and immune system across non-human primates and non-primate species, aiming to understand their evolutionary processes. We examined 1360 human genes in 32 primate species (including human) and 4 non-primate species, with a comparison set of 295 randomly selected genes and 382 genes from distinct top-level gene families.

Our findings show that brain highly expressed genes are more evolutionarily preserved than those related to the immune system or random genes. This difference between brain genes and immune-related genes is not likely due to differences in their expression breadth. Moreover, if a gene is highly expressed both in the brain and immune system, it is more likely to have an origin time similar to other brain highly expressed genes, indicating the origins of the duel expressed genes may be associated with the brain instead of the immune system.

For those brain genes that are found in certain primate clades but not others, they are more likely newly evolved rather than eliminated from some species. Furthermore, we found that certain brain genes, emerging in primates and preserved or lost in specific primate clades, exhibit broad tissue expression or are confined to specific brain regions like the cerebellum or cerebral cortex.

## Supporting information

S1 File Supporting figure and tables. (PDF)
