# Supplementary material for: Comparative genomics of human brain and immune gene preservation across species
Source: PLoS One. 2026 May 11;21(5):e0348713. doi: 10.1371/journal.pone.0348713 (PMC13160339; doi:10.1371/journal.pone.0348713)
Supplement: S1 File — (PDF) [file pone.0348713.s001.pdf]

Supplementary Material

This Supplementary Material contains tables generated based on more relaxed thresholds compared to the main text: (1) 60% BLAST identity and 60% BLAST query coverage per subject on all species in this study (i.e., 31 primate species and 4 non-primate species), and (2) 60% BLAST identity and 60% BLAST query coverage per subject on 4 non-primate species only. These different thresholds were applied to (1) demonstrate the resilience of analysis results under different threshold, and (2) account for further evolutionary distance of non-primate species from human compared to primate species.

Note that results not affected by the relaxed thresholds (e.g., how many tissues are certain genes highly expressed in) are not included in this Supplementary Material.

At the looser sequence similarity cutoff, some hits may correspond to distant members of the same gene family rather than direct orthologs. However, even distant sequence similarity implies a shared ancestral sequence predating the relevant species divergence. The effect of loosening the threshold is that fewer genes are classified as primate-specific or clade-absent, as expected. The relative pattern between brain and immune gene sets remains consistent across both cutoffs, suggesting that the findings are not sensitive to this distinction.

This Supplementary Material also includes ontology enrichment results for brain, immune, and common genes, repeated subsampling analyses performed to assess whether the larger size of $\mathcal{B}$ influenced the statistical results, as well as statistical tests for the cross-database validation.

Results with Relaxed Threshold Applied to All Species

Table S1 represents the distribution of $\mathcal{B}$, $\mathcal{I}$, $\mathcal{C}$, $\mathcal{R}$, and $\mathcal{F}$ among 31 non-human primates and 4 non-primate species. Overall, the genes highly expressed in the brain ($\mathcal{B}$ and $\mathcal{C}$) have lower primate specific ratios, compared to control gene sets and genes highly expressed in the immune system, but not in the brain.

Table S1: The distribution of $\mathcal{B}$, $\mathcal{I}$, $\mathcal{C}$, $\mathcal{R}$, and $\mathcal{F}$ among 31 non-human primates and 4 non-primate species. We use N to represent the total number of genes of a gene set, J to represent the total number of genes out from each gene set that were identified in all 31 non-human primates, K to represent the total number of genes out from each gene set that were identified in a proper subset of 31 non-human primates, L to represent the total number of genes out from each gene set that were identified in none of 4 non-primate species. We call $\alpha = \frac{L}{N}$ the "primate specific ratio".

**Gene Set**	**Total number of genes N**	**Number of genes identified in all 31 primates J**	**Number of genes identified in a proper subset of the 31 primates K**	**Number of genes identified in none of the 4 non-primate species L**	**Primate specific ratio $\frac{L}{N}$**
$\mathcal{B}$	1026	659	367	13	0.01
$\mathcal{I}$	587	370	217	17	0.03
$\mathcal{C}$	252	162	90	4	0.02
$\mathcal{R}$	300	171	129	17	0.06
$\mathcal{F}$	375	246	129	15	0.04

Fisher's exact test results presented in Table S2 show that both $\mathcal{B}$ and $\mathcal{C}$ have lower primate-specific ratios than $\mathcal{R}$, and that $\mathcal{B}$ also has a lower primate-specific ratio than $\mathcal{F}$. By contrast, no statistically significant differences are detected between $\mathcal{I}$ and either $\mathcal{R}$ or $\mathcal{F}$, nor between $\mathcal{C}$ and $\mathcal{F}$.

Table S2: Contingency tables and Fisher's exact test results for pairwise comparisons. Positive cases are primate-specific genes, while negative cases are genes that are not primate specific. Statistical significance is denoted by asterisks: results with p-values less than 0.01 are indicated by two asterisks (**), whereas results with p-values less than 0.05 are indicated by one asterisk (*). An odds ratio larger than 1 suggests the group is more likely to be primate specific compared to $\mathcal{R}$ or $\mathcal{F}$.

Comparison	Group	Positive	Negative	Total	Odds Ratio	Fisher's p-value
$\mathcal{B}$ vs. $\mathcal{R}$	$\mathcal{B}$<br>$\mathcal{R}$	13<br>17	1014<br>283	1026<br>300	0.214	4.595e-05**
$\mathcal{I}$ vs. $\mathcal{R}$	$\mathcal{I}$<br>$\mathcal{R}$	17<br>17	570<br>283	587<br>300	0.496	0.0346*
$\mathcal{C}$ vs. $\mathcal{R}$	$\mathcal{C}$<br>$\mathcal{R}$	4<br>17	248<br>283	252<br>300	0.269	0.0096**
$\mathcal{B}$ vs. $\mathcal{F}$	$\mathcal{B}$<br>$\mathcal{F}$	13<br>15	1014<br>360	1026<br>375	0.308	0.0021**
$\mathcal{I}$ vs. $\mathcal{F}$	$\mathcal{I}$<br>$\mathcal{F}$	17<br>15	570<br>360	587<br>375	0.716	0.226
$\mathcal{C}$ vs. $\mathcal{F}$	$\mathcal{C}$<br>$\mathcal{F}$	4<br>15	248<br>360	252<br>375	0.387	0.064
$\mathcal{F}$ vs. $\mathcal{R}$	$\mathcal{F}$<br>$\mathcal{R}$	15<br>17	360<br>283	375<br>300	0.694	0.203

Table S3 presents the results of Cohen's h to further assess whether $\mathcal{C}$'s primate-specific proportion more closely resembles that of $\mathcal{B}$ or $\mathcal{I}$. This result also indicates that the genes highly expressed in both the brain and immune system is more similar in their evolutionary origin to brain genes rather than immune-related genes.

Group	Positive (%)	Total n
$\mathcal{B}$	1.3	1026
$\mathcal{I}$	2.8	587
$\mathcal{C}$	1.6	252
**Comparison**	**Cohen's h**	**Interpretation**
$\mathcal{C}$ vs $\mathcal{B}$	0.026	Small
$\mathcal{C}$ vs $\mathcal{I}$	-0.086	Small

Table S3: Proportion of positive or negative outcomes for each group, and Cohen's h values for pairwise comparisons between $\mathcal{C}$ and the other two groups: $\mathcal{B}$ and $\mathcal{I}$. Here, positive cases are primate-specific genes, while negative cases are genes that are not primate specific. A higher absolute Cohen's h indicates a greater difference in proportions.

Table S4 presents a detailed summary of gene absences across primate clades. In Table S5, we use Fisher's exact test to compare the proportion of positive outcomes (i.e., the genes absent from large clades or small clades) in $\mathcal{B}$, $\mathcal{I}$, and $\mathcal{C}$ to that of $\mathcal{R}$ or $\mathcal{F}$, as well as a comparison in between the two cotrol sets $\mathcal{R}$ and $\mathcal{F}$. The analysis shows that, in both the large and small primate clades, genes highly expressed in the brain or immune system ($\mathcal{B}$, $\mathcal{I}$, and $\mathcal{C}$) tend to have lower clade-absent ratios compared to randomly selected human genes ($\mathcal{R}$) and human genes from distinct gene families ($\mathcal{F}$).

Table S4: The absence of genes from $\mathcal{B}$, $\mathcal{I}$, $\mathcal{C}$, $\mathcal{R}$, and $\mathcal{F}$ in primate clades within 31 non-human primates. Within the genes exist in at least one of the 31 primate species, we use N to represent the total number of genes of a gene set, P to represent the total number of genes that are absent from large clades, and Q to represent the total number of genes that are absent from small clades. We call $\beta = \frac{P}{N}$ and $\gamma = \frac{Q}{N}$ "large clade absent ratio" and "small clade absent ratio" respectively.

**Gene Set**	**Total number of genes** N	**Number of genes absent from large clades** P	**Number of genes absent from small clades** Q	**Large clade absent ratio** $\frac{P}{N}$	**Small clade absent ratio** $\frac{Q}{N}$
$\mathcal{B}$	1026	5	13	0.005	0.013
$\mathcal{I}$	587	7	8	0.012	0.014
$\mathcal{C}$	252	1	5	0.004	0.020
$\mathcal{R}$	300	14	16	0.047	0.053
$\mathcal{F}$	375	13	9	0.035	0.024

Table S5: Contingency tables and Fisher's exact test results for pairwise comparisons. Positive cases are primate-specific genes, while negative cases are genes that are not primate specific. Statistical significance is denoted by asterisks: results with p-values less than 0.01 are indicated by two asterisks (**), whereas results with p-values less than 0.05 are indicated by one asterisk (*). An odds ratio larger than 1 suggests the group is more likely to be primate specific compared to $\mathcal{R}$ or $\mathcal{F}$.

**Comparison**	**Group**	**Positive**	**Negative**	**Total**	**Odds Ratio**	**Fisher's p-value**
$\mathcal{B}$ vs. $\mathcal{R}$	$\mathcal{B}$	5	1021	1026	0.1000	2.706e-06**
	$\mathcal{R}$	14	286	300		
$\mathcal{I}$ vs. $\mathcal{R}$	$\mathcal{I}$	7	580	587	0.2466	0.0019**
	$\mathcal{R}$	14	286	300		
$\mathcal{C}$ vs. $\mathcal{R}$	$\mathcal{C}$	1	251	252	0.08139	0.0013**
	$\mathcal{R}$	14	286	300		
$\mathcal{B}$ vs. $\mathcal{F}$	$\mathcal{B}$	5	1021	1026	0.1364	6.665e-05**
	$\mathcal{F}$	13	362	375		
$\mathcal{I}$ vs. $\mathcal{F}$	$\mathcal{I}$	7	580	587	0.3361	0.0157*
	$\mathcal{F}$	13	362	375		
$\mathcal{C}$ vs. $\mathcal{F}$	$\mathcal{C}$	1	251	252	0.1109	0.0073**
	$\mathcal{F}$	13	362	375		
$\mathcal{F}$ vs. $\mathcal{R}$	$\mathcal{F}$	13	362	375	0.7336	0.2755
	$\mathcal{R}$	14	286	300		

Results with Relaxed Threshold Applied to Non-Primate Species Only

Table S6 represents the distribution of $\mathcal{B}$, $\mathcal{I}$, $\mathcal{C}$, $\mathcal{R}$, and $\mathcal{F}$ among 31 non-human primates and 4 non-primate species. Overall, the genes highly expressed in the brain ($\mathcal{B}$ and $\mathcal{C}$) have lower primate specific ratios, compared to control gene sets and genes highly expressed in the immune system, but not in the brain.

Table S6: The distribution of $\mathcal{B}$, $\mathcal{I}$, $\mathcal{C}$, $\mathcal{R}$, and $\mathcal{F}$ among 31 non-human primates and 4 non-primate species. We use N to represent the total number of genes of a gene set, J to represent the total number of genes out from each gene set that were identified in all 31 non-human primates, K to represent the total number of genes out from each gene set that were identified in a proper subset of 31 non-human primates, L to represent the total number of genes out from each gene set that were identified in none of 4 non-primate species. We call $\alpha = \frac{L}{N}$ the "primate specific ratio".

**Gene Set**	**Total number of genes** N	**Number of genes identified in all 31 primates** J	**Number of genes identified in a proper subset of the 31 primates** K	**Number of genes identified in none of the 4 non-primate species** L	**Primate specific ratio** $\frac{L}{N}$
$\mathcal{B}$	1019	352	667	11	0.01
$\mathcal{I}$	586	192	394	15	0.03
$\mathcal{C}$	245	78	167	3	0.01
$\mathcal{R}$	295	73	222	17	0.06
$\mathcal{F}$	369	135	234	15	0.04

Fisher's exact test results presented in Table S7 show that both $\mathcal{B}$ and $\mathcal{C}$ have lower primate-specific ratios than $\mathcal{R}$, and that $\mathcal{B}$ also has a lower primate-specific ratio than $\mathcal{F}$. By contrast, no statistically significant differences are detected between $\mathcal{I}$ and either $\mathcal{R}$ or $\mathcal{F}$, nor between $\mathcal{C}$ and $\mathcal{F}$.

Table S8 presents the results of Cohen's h to further assess whether $\mathcal{C}$'s primate-specific proportion more closely resembles that of $\mathcal{B}$ or $\mathcal{I}$. This result also indicates that the genes highly expressed in both the brain and immune system is more similar in their evolutionary origin to brain genes rather than immune-related genes.

Ontology Enrichment for Brain, Immune, and Common genes

Figure 1 shows the enrichment results using using Gene Ontology biological process (GO BP) terms and Reactome pathways. The observed enrichment profiles were broadly in agreement with the expected brain and immune-related functions, supporting the biological relevance of these gene sets.

Subsampling analysis to assess the effect of $\mathcal{B}$ gene set size

To assess whether the larger size of $\mathcal{B}$ influenced the statistical results, we performed 1000 repeated subsampling analyses in which random subsets of $\mathcal{B}$ were drawn without replacement to match the size of $\mathcal{I}$ ($n = 586$). We denote these size-matched subsets by $\mathcal{B}^{(s)}$. For each subsample, we recalculated the same summary counts used in the main analysis, including the primate-specific count (L) and the large-clade absent count (P), and then repeated the corresponding pairwise Fisher's exact tests. For the primate-specific analysis, Cohen's h was also recomputed. The resulting tables, Table S9 and Table S10, report the median odds ratio (and, where applicable, median Cohen's h), together with the percentage of resamples in which the odds ratio remained in the original direction and the percentage with $p < 0.05$.

For the primate-specific analysis, repeated subsampling of $\mathcal{B}$ to match the size of $\mathcal{I}$ yielded results consistent with the original conclusions. Across resamples, $\mathcal{B}^{(s)}$ remained lower than $\mathcal{I}$, $\mathcal{R}$, and $\mathcal{F}$, with median odds ratios of 0.374, 0.317, and 0.499, respectively, and negative median Cohen's h values in all three comparisons. In each case, the odds ratio remained in the original direction in 100% of resamples, and all resamples were significant at $p < 0.05$. By contrast, the comparison between $\mathcal{B}^{(s)}$ and $\mathcal{C}$ was weaker, with a median odds ratio closer to 1 (0.737),

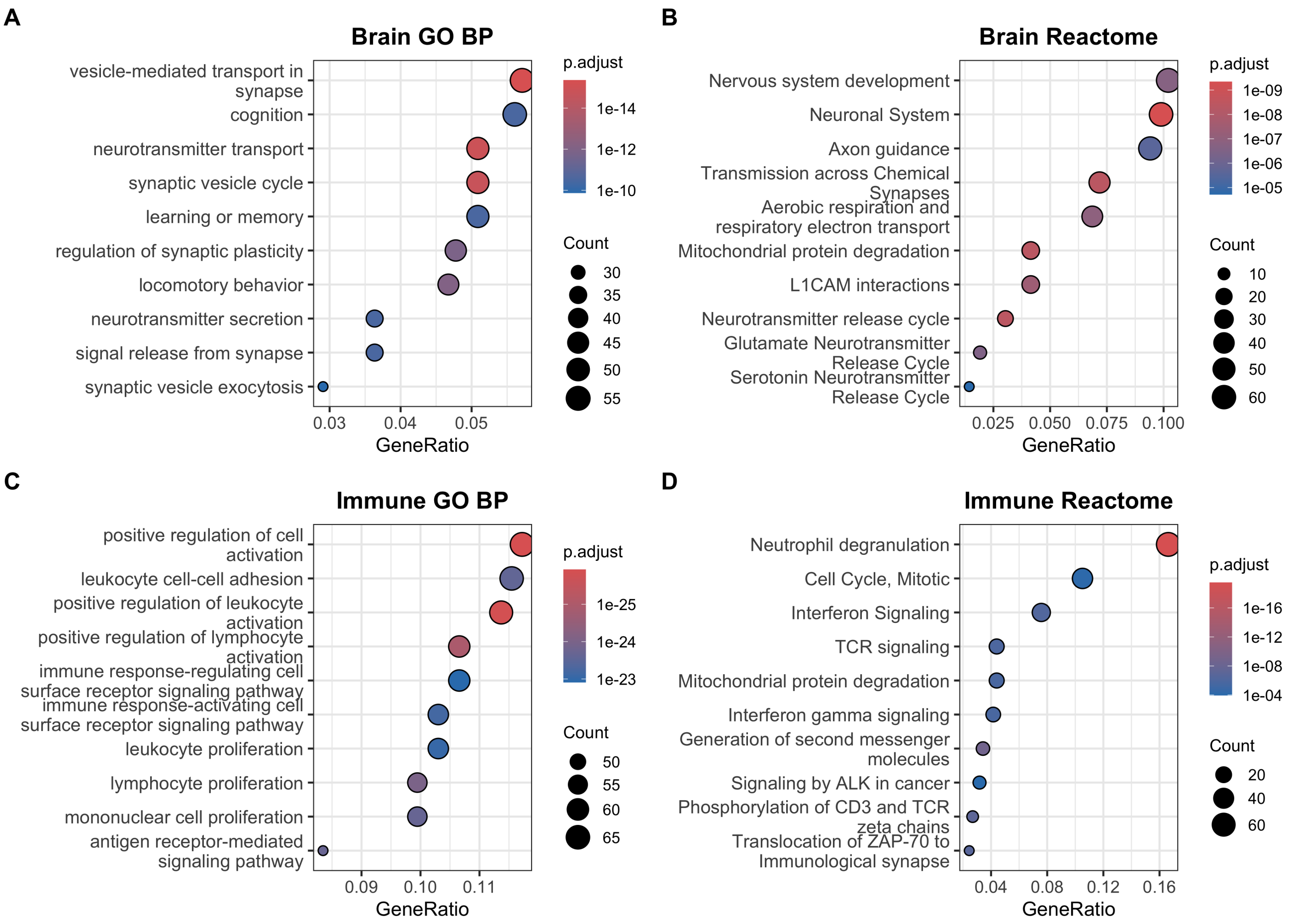

Figure 1: Functional enrichment of genes highly expressed in brain and immune-related tissues. Over-representation analysis (ORA) was performed separately for the brain gene set ($\mathcal{B}$) and the immune gene set ($\mathcal{I}$) using Gene Ontology biological process (GO BP) terms and Reactome pathways. The dotplots show the top 10 enriched categories for (A) brain GO BP, (B) brain Reactome, (C) immune GO BP, and (D) immune Reactome. Dot size represents gene count, and color represents adjusted p-value.

Table S7: Contingency tables and Fisher's exact test results for pairwise comparisons. Positive cases genes absent from large clades. Statistical significance is denoted by asterisks: results with p-values less than 0.01 are indicated by two asterisks (**), whereas results with p-values less than 0.05 are indicated by one asterisk (*). An odds ratio larger than 1 suggests the group is more likely to be absent from large clades compared to $\mathcal{R}$ or $\mathcal{F}$.

Comparison	Group	Positive	Negative	Total	Odds Ratio	Fisher's p-value
$\mathcal{B}$ vs. $\mathcal{R}$	$\mathcal{B}$<br>$\mathcal{R}$	11<br>17	1008<br>278	1019<br>295	0.1785	1.159e-05**
$\mathcal{I}$ vs. $\mathcal{R}$	$\mathcal{I}$<br>$\mathcal{R}$	15<br>17	571<br>278	586<br>295	0.4296	0.0155*
$\mathcal{C}$ vs. $\mathcal{R}$	$\mathcal{C}$<br>$\mathcal{R}$	3<br>17	242<br>278	245<br>295	0.2027	0.0039**
$\mathcal{B}$ vs. $\mathcal{F}$	$\mathcal{B}$<br>$\mathcal{F}$	11<br>15	1008<br>354	1019<br>369	0.2575	0.0007**
$\mathcal{I}$ vs. $\mathcal{F}$	$\mathcal{I}$<br>$\mathcal{F}$	15<br>15	571<br>354	586<br>369	0.6200	0.1344
$\mathcal{C}$ vs. $\mathcal{F}$	$\mathcal{C}$<br>$\mathcal{F}$	3<br>15	242<br>354	245<br>369	0.2926	0.0315*
$\mathcal{F}$ vs. $\mathcal{R}$	$\mathcal{F}$<br>$\mathcal{R}$	15<br>17	354<br>278	369<br>295	0.6929	0.2022

Group	Positive (%)	Total n
$\mathcal{B}$	1.1	1019
$\mathcal{I}$	2.5	586
$\mathcal{C}$	1.2	245
**Comparison**	**Cohen's h**	**Interpretation**
$\mathcal{C}$ vs $\mathcal{B}$	0.013	Small
$\mathcal{C}$ vs $\mathcal{I}$	-0.097	Small

Table S8: Proportion of positive or negative outcomes for each group, and Cohen's h values for pairwise comparisons between $\mathcal{C}$ and the other two groups: $\mathcal{B}$ and $\mathcal{I}$. Here, positive cases are primate-specific genes, while negative cases are genes that are not primate specific. A higher absolute Cohen's h indicates a greater difference in proportions.

a small median Cohen's h (-0.090), and statistical significance in only 1.6% of resamples. These results indicate that, with respect to the primate-specific ratio, $\mathcal{C}$ remains more similar to $\mathcal{B}$ than to $\mathcal{I}$, while $\mathcal{B}$ remains distinct from $\mathcal{I}$, $\mathcal{R}$, and $\mathcal{F}$.

For the large-clade absence analysis, the same overall pattern was observed. After repeated subsampling, $\mathcal{B}^{(s)}$ remained lower than $\mathcal{I}$, $\mathcal{R}$, and $\mathcal{F}$, with median odds ratios of 0.413, 0.258, and 0.396, respectively; in all three comparisons, the odds ratio remained in the original direction in 100% of resamples and all resamples were significant at $p < 0.05$. In contrast, the comparison between $\mathcal{B}^{(s)}$ and $\mathcal{C}$ was weaker, with a median odds ratio close to 1 (0.955), the original direction retained in only 63.9% of resamples, and no resample reaching statistical significance. Thus, also for the large-clade absent ratio, $\mathcal{C}$ remains more similar to $\mathcal{B}$ than to $\mathcal{I}$, whereas $\mathcal{B}$ remains distinct from $\mathcal{I}$, $\mathcal{R}$, and $\mathcal{F}$.

Statistics Tests for Cross-database Validation

Fisher's exact test results presented in Table S11 show that both $\mathcal{B}^*$ and $\mathcal{C}^*$ have lower primate-specific ratios than $\mathcal{R}$, and that $\mathcal{B}^*$ also has a lower primate-specific ratio than $\mathcal{F}$. By contrast, no statistically significant differences

Table S9: Summary of repeated subsampling analyses for pairwise comparisons based on the primate-specific ratio. $\mathcal{B}^{(s)}$ denotes a randomly sampled subset of $\mathcal{B}$ matched in size to $\mathcal{I}$. For each subsample, Fisher's exact test and Cohen's h were calculated using the same procedure as in the main analysis. The table reports the median odds ratio (OR), the median Cohen's h, the percentage of subsamples in which the OR was in the same direction as in the original analysis (i.e., OR < 1 when the original OR was < 1, and vice versa), and the percentage of subsamples with $p < 0.05$.

**Comparison**	**Median OR**	**Median Cohen's h**	**Resamples with OR in original direction (%)**	**Resamples with $p < 0.05$ (%)**
$\mathcal{B}^{(s)}$ vs $\mathcal{I}$	0.374	-0.331	100.0	100.0
$\mathcal{B}^{(s)}$ vs $\mathcal{R}$	0.317	-0.399	100.0	100.0
$\mathcal{B}^{(s)}$ vs $\mathcal{F}$	0.499	-0.221	100.0	100.0
$\mathcal{B}^{(s)}$ vs $\mathcal{C}$	0.737	-0.090	100.0	1.6

Table S10: Summary of repeated subsampling analyses for pairwise comparisons based on the large-clade absent ratio. $\mathcal{B}^{(s)}$ denotes a randomly sampled subset of $\mathcal{B}$ matched in size to $\mathcal{I}$. For each subsample, Fisher's exact test and Cohen's h were calculated using the same procedure as in the main analysis. The table reports the median odds ratio (OR), the percentage of subsamples in which the OR was in the same direction as in the original analysis (i.e., OR < 1 when the original OR was < 1, and vice versa), and the percentage of subsamples with $p < 0.05$.

**Comparison**	**Median OR**	**Resamples with OR in original direction (%)**	**Resamples with $p < 0.05$ (%)**
$\mathcal{B}^{(s)}$ vs $\mathcal{I}$	0.413	100.0	100.0
$\mathcal{B}^{(s)}$ vs $\mathcal{R}$	0.258	100.0	100.0
$\mathcal{B}^{(s)}$ vs $\mathcal{F}$	0.396	100.0	100.0
$\mathcal{B}^{(s)}$ vs $\mathcal{C}$	0.955	63.9	0.0

are detected between $\mathcal{I}^*$ and either $\mathcal{R}$ or $\mathcal{F}$, nor between $\mathcal{C}^*$ and $\mathcal{F}^*$.

Table S11 presents the results of Cohen's h to further assess whether $\mathcal{C}^*$'s primate-specific proportion more closely resembles that of $\mathcal{B}^*$ or $\mathcal{I}^*$. This result also indicates that the genes highly expressed in both the brain and immune system is more similar in their evolutionary origin to brain genes rather than immune-related genes.

Fisher's exact test results presented in Table S13 show that both $\mathcal{B}^*$ and $\mathcal{C}^*$ have lower clade-absent ratios than $\mathcal{R}$, and that $\mathcal{B}^*$ also has a lower primate-specific ratio than $\mathcal{F}$. By contrast, no statistically significant differences are detected between $\mathcal{I}^*$ and either $\mathcal{R}$ or $\mathcal{F}$, nor between $\mathcal{C}^*$ and $\mathcal{F}^*$.

Table S11: Contingency tables and Fisher's exact test results for pairwise comparisons among $\mathcal{B}^*$, $\mathcal{I}^*$, $\mathcal{C}^*$, $\mathcal{R}$, and $\mathcal{F}$. Positive cases are primate-specific genes, while negative cases are genes that are not primate specific. Statistical significance is denoted by asterisks: results with p-values less than 0.01 are indicated by two asterisks (**), whereas results with p-values less than 0.05 are indicated by one asterisk (*). An odds ratio larger than 1 suggests the group is more likely to be primate specific compared to $\mathcal{R}$ or $\mathcal{F}$.

**Comparison**	**Group**	**Positive**	**Negative**	**Total**	**Odds Ratio**	**Fisher's p-value**
$\mathcal{B}$ vs. $\mathcal{R}$	$\mathcal{B}$	25	511	536	0.1698	2.457e-14**
	$\mathcal{R}$	66	229	295		
$\mathcal{I}$ vs. $\mathcal{R}$	$\mathcal{I}$	52	204	256	0.8844	0.3147
	$\mathcal{R}$	66	229	295		
$\mathcal{C}$ vs. $\mathcal{R}$	$\mathcal{C}$	4	70	74	0.1983	0.0002662**
	$\mathcal{R}$	66	229	295		
$\mathcal{B}$ vs. $\mathcal{F}$	$\mathcal{B}$	25	511	536	0.2678	3.364e-08**
	$\mathcal{F}$	57	312	369		
$\mathcal{I}$ vs. $\mathcal{F}$	$\mathcal{I}$	52	204	256	1.395	0.9533
	$\mathcal{F}$	57	312	369		
$\mathcal{C}$ vs. $\mathcal{F}$	$\mathcal{C}$	4	70	74	0.3128	0.01224*
	$\mathcal{F}$	57	312	369		
$\mathcal{F}$ vs. $\mathcal{R}$	$\mathcal{F}$	57	312	369	0.6339	0.01474*
	$\mathcal{R}$	66	229	295		

**Group**	**Positive (%)**	**Total n**
$\mathcal{B}^*$	4.7	536
$\mathcal{I}^*$	20.3	256
$\mathcal{C}^*$	5.4	74
**Comparison**	**Cohen's h**	**Interpretation**
$\mathcal{C}^*$ vs $\mathcal{B}^*$	0.034	Small
$\mathcal{C}^*$ vs $\mathcal{I}^*$	-0.466	Small

Table S12: Proportion of positive outcomes for each group, and Cohen's h values for pairwise comparisons between $\mathcal{C}^*$ and the other two groups: $\mathcal{B}^*$ and $\mathcal{I}^*$. Here, positive cases are primate-specific genes. A higher absolute Cohen's h indicates a greater difference in proportions.

Table S13: Contingency tables and Fisher's exact test results for pairwise comparisons among $\mathcal{B}^*$, $\mathcal{I}^*$, $\mathcal{C}^*$, $\mathcal{R}$, and $\mathcal{F}$. Statistical significance is indicated by asterisks: p-values less than 0.01 are denoted by two asterisks (**), and p-values less than 0.05 by one asterisk (*). An odds ratio greater than 1 indicates that the gene set in the numerator is more likely to be absent from large clades than the reference set in the comparison.

**Comparison**	**Group**	**Positive**	**Negative**	**Total**	**Odds Ratio**	**Fisher's p-value**
$\mathcal{B}$ vs. $\mathcal{R}$	$\mathcal{B}$<br>$\mathcal{R}$	17<br>54	519<br>241	536<br>295	0.1462	3.425e-13**
$\mathcal{I}$ vs. $\mathcal{R}$	$\mathcal{I}$<br>$\mathcal{R}$	38<br>54	218<br>241	256<br>295	0.7779	0.1656
$\mathcal{C}$ vs. $\mathcal{R}$	$\mathcal{C}$<br>$\mathcal{R}$	3<br>54	71<br>241	74<br>295	0.1886	0.0008365**
$\mathcal{B}$ vs. $\mathcal{F}$	$\mathcal{B}$<br>$\mathcal{F}$	17<br>47	519<br>322	536<br>369	0.2244	4.124e-08**
$\mathcal{I}$ vs. $\mathcal{F}$	$\mathcal{I}$<br>$\mathcal{F}$	38<br>47	218<br>322	256<br>369	1.194	0.8093
$\mathcal{C}$ vs. $\mathcal{F}$	$\mathcal{C}$<br>$\mathcal{F}$	3<br>47	71<br>322	74<br>369	0.2895	0.01804*
$\mathcal{F}$ vs. $\mathcal{R}$	$\mathcal{F}$<br>$\mathcal{R}$	47<br>54	322<br>241	369<br>295	0.6514	0.03059*
